# Ultra-High Capacity Optical Satellite Communication System Using PDM-256-QAM and Optical Angular Momentum Beams

**DOI:** 10.3390/s23020786

**Published:** 2023-01-10

**Authors:** Shippu Sachdeva, Simarpreet Kaur, Romisha Arora, Manoj Sindhwani, Krishan Arora, Woong Cho, Gyanendra Prasad Joshi, Ill Chul Doo

**Affiliations:** 1 School of Electronics and Electrical Engineering, Lovely Professional University, Phagwara 144411, India; 2 Department of Electronics and Communication, Chandigarh University, Mohali 140413, India; 3 Department of Electronics and Communication, Manav Rachna International Institute of Research and Studies, Faridabad 121004, India; 4 Department of Software Convergence, Daegu Catholic University, Gyeongsan 38430, Republic of Korea; 5 Department of Computer Science and Engineering, Sejong University, Seoul 05006, Republic of Korea; 6 Artificial Intelligence Education, Hankuk University of Foreign Studies, Dongdaemun-gu, Seoul 02450, Republic of Korea

**Keywords:** OSC, OAM, QAM, DSP, TLBC

## Abstract

Twisted light beams such as optical angular momentum (OAM) with numerous possible orthogonal states have drawn the prodigious contemplation of researchers. OAM multiplexing is a futuristic multi-access technique that has not been scrutinized for optical satellite communication (OSC) systems thus far, and it opens up a new window for ultra-high-capacity systems. This paper presents the 4.8 Tbps (5 wavelengths × 3 OAM beams × 320 Gbps) ultra-high capacity OSC system by incorporating polarization division multiplexed (PDM) 256-Quadrature amplitude modulation (256-QAM) and OAM beams. To realize OAM multiplexing, Laguerre Gaussian (LG) transverse mode profiles such as LG00, LG140, and LG400 were used in the proposed study. The effects of the receiver’s digital signal processing (DSP) module were also investigated, and performance improvement was observed using DSP for its potential to compensate for the effects of dispersion, phase errors, and nonlinear effects using the blind phase search (BPS), Viterbi phase estimation (VPE), and the constant modulus algorithm (CMA). The results revealed that the proposed OAM-OSC system successfully covered the 22,000 km OSC link distance and, out of three OAM beams, fundamental mode LG00 offered excellent performance. Further, a detailed comparison of the proposed system and reported state-of-the-art schemes was performed.

## 1. Introduction

**Figure 1:**
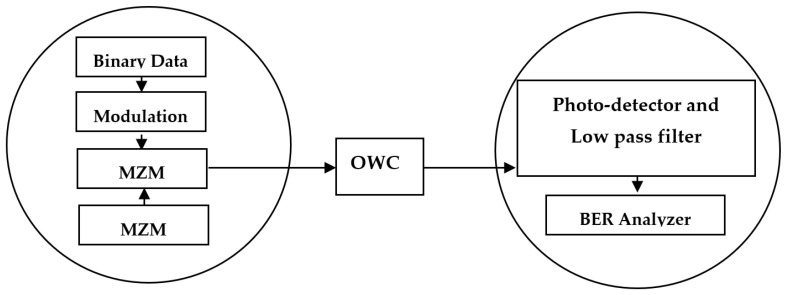
General representation of OSC link.

With the proliferation of multimedia services and internet applications such as high definition (HD) live streaming, HD video conferencing, voice over IP (VoIP), 4/8 K video transmission, and recently introduced virtual reality technology, i.e., metaverse, contemporary data transmission techniques are experiencing peer pressure due to explosive demands for wide-bandwidth [1]. Radio-frequency communication (RFC) is recognized as a mature through-hole technology for a wide range of terrestrial and space applications [2]. However, RFC does not efficiently support high-speed fifth-generation (5G) and sixth-generation (6G) services due to severe constraints such as bandwidth scarcity, multipath fading, predominant amplitude degradation, vulnerability to security breaches, and the requirement for data transmission licenses [3]. Twisted light-based communication (LBC) has experienced a paradigm shift in data transmission under guided or unguided wireless mediums due to its narrow light beamwidth, unlicensed operations, improved security, high power efficiency, lower amplitude degradations, absence of electromagnetic interference (EMI), and the potential to support high-speed networks [4,5,6]. There are three types of LBC applications such as optical indoor communication (OIC) (short reach up to 5 m) [7], optical terrestrial communication (OTC) (1–30 km) [8], and optical satellite communication (OSC) (ultra long reach up to 80,000 km) [9]. Over the years, there has been a prominent upsurge in the satellite constellations in space, and it is estimated that satellite deployment will grow by 1000% in the next decade. Therefore, OSC is the next frontier and offers rigorous development in broadcasting, navigation, communication, weather forecasting, etc. [10]. The block diagram of the OSC system between two satellites is depicted in Figure 1.

Satellites can be placed in any of the three available earth orbits depending upon the distance from the earth, such as low satellite orbit (LSO) (≤2000 km), medium satellite orbit (MSO) (≤20,000 km), and high earth orbit (HSO) (≥35,000 km) [11,12,13]. The orbiting time of LSO and MSO around the earth is 2–4 and 5–12 h, respectively. Deep space communication refers to interplanetary links that can extend more than 1,500,000 km above the earth. The performance of OSC systems depends on transmitter power, the distances between satellites, transmitter and receiver efficiency, atmospheric losses, line of sight (LOS), pointing and tracking errors (PTE), telescope aperture size, and modulation/line coding/advanced modulation format/multi-level modulations, etc. Background noise, satellite platform vibrations, and PTE are the most dominant impediments in OSC. High-capacity OSC systems can be realized by incorporating multiple wavelengths (WDM), different time slots (time division), diverse codes (optical code division), and distinct modes (mode division multiplexing) (MDM) [14,15,16].

### 1.1. Development of OSC System

The European Space Agency (ESA) demonstrated the first OSC link at 50 Mbps between ARTEMIS and SPOT-4 [17]. In [18], an LSO-based 5500 km OSC link between NFIRE and SAR-X at 5.5 Gbps was reported in [18] in 2008. The data rate was increased to 5.6 Gbps in TerraSAR-X satellites using binary shift keying (BPSK) and covered 6000 km of link length [19]. In 2012, a multilevel quadrature phase shift keying (QPSK) modulation was first demonstrated over a 9352 km OSC link distance for the binary data speed of 100 Gbps [20]. QPSK has capacity doubling advantages because of using two binary bits per symbol and hence reduces the strain on the electronic binary data generator. Non-return to zero (NRZ) and return to zero (RZ) are the primary modulation formats and are widely incorporated in the OSC system due to their simple architecture and reasonable cost [21,22,23,24,25,26,27,28]. However, these formats are not bandwidth and power efficient for optical communication systems. Carrier spectrum efficiency improvement is reported by the emergence of three different advanced pulse formats (APF), such as carrier spectrum RZ (CSRZ), modified duo-binary (MDRZ), and DRZ [29]. Despite the higher spectral efficiency, these APFs failed to support data rates higher than 40 Gbps per channel. As a result, differential phase shift keying (DPSK) was investigated in the OSC systems to eliminate the need for fixed thresholding at the receiver, as opposed to NRZ/RZ, and to provide better power efficiency [30]. DPSK has a higher probability of errors in the received bits because it requires a pair of two bits for decoding, and sometimes an error in the first bit also causes an error in the second bit. Phase shifting in the data offers higher tolerance to pulse broadening and reduces interchannel interference (ISI). Differential quadrature PSK is also investigated in 50,000 km OSC systems at 200 Gbps and has 0, π, +π/2, and −π/2 phase shifts [31]. High noise and phase errors make DQPSK less popular in OSC systems. For the MSO and HSO communications, multilevel modulations play a remarkable role in the OSC system due to their high power efficiency, improved receiver sensitivity, narrow spectrum, and prolonged distances. Some of the most widely used multilevel modulations are orthogonal frequency division multiplexing (OFDM), QPSK, and quadrature amplitude modulation (QAM). OFDM has improved resistance to ISI and high spectral efficiency but has the limitation of a high peak-to-average noise ratio (PAPR). In [32], a radio frequency-enabled 35,000 km OSC system with OFDM and 4-QAM at 20 Gbps was presented. In [33], 4-QAM in OFDM was replaced with DPSK encoding, and it was perceived that the OSC system covered 20,000 km at 10 Gbps. Coherent optical QPSK is also an attractive spectral-efficient and highly noise-resistant modulation for OSC systems. An OSC distance of 42,500 km has been achieved by incorporating CO-QPSK and digital signal processing at 40 Gbps [34]. The third multilevel modulation is QAM, and it comes in different variants, such as 8-QAM to 512-QAM. It has greater power efficiency than QPSK due to the higher number of bits per symbol. CO-16-QAM was investigated in the OSC system and offered 45,000 km at 1 Tbps capacity operating in the conventional band (C-Band) [35].

The polarization dimension is an important property of light among other dimensions such as frequency, space, and time, but it has yet to be extensively explored to obtain ultra-high data rates. The hybridization of multilevel modulations with different states of polarizations (SOPs) has opened a new window for high-capacity optical systems [36]. In the literature, the hybridization of OFDM, QAM, and QPSK has been reported with different SOPs, termed PDM, allowing lower-speed electronics, high receiver sensitivity, and long-range OSC links. However, these systems are vulnerable to pulse width broadening (PWB), phase errors, nonlinear effects, other noises, etc. Therefore, researchers suggested a remarkable signal processing technique for high-performance and least EVMs, i.e., DSP, compensating for the effects of phase mismatching, PWB, Kerr’s effects, and other noises using different algorithms. Most OSC systems now incorporate PDM and multilevel modulations for high-speed and long-distance transmissions.

### 1.2. Hybrid Multilevel Modulations and PDM/MDM Enabled OSC Systems

Ultra-high speeds of several Tbps can be achieved with the deployment of PDM in multilevel modulations. An ultra-long-range 60,000 km OSC link was proposed employing PDM in CO-OFDM at 400 Gbps [37]. Further, at the same speed, a security-improved PDM-enabled 25,000 km long OSC link was reported in [38] by employing spectral amplitude codes (SAC). A 40,000 km gap between two satellites at 160 Gbps over a single channel PDM-enabled QPSK was demonstrated in [39]. A high capacity 16 × 100 Gbps OSC system was proposed using PDM-QPSK in CO-OFDM and obtained a 15,600 km total reach [40]. Further, a futuristic and potential multiplexing technique incorporating different intensity profiles of laser light was introduced in OSC systems for high-speed and ultra-long reach links. Different 64-intensity profiles of light were generated from azimuthal (*l*), and radial numbers (*m*) of EM waves in [41] for a 40 Gbps (maximum) OSC link incorporating differential QPSK, Manchester, and DPSK modulations. Results indicated that DQPSK performed far better than DPSK and Manchester coding over 3750 km of OSC link distance. OFDM modulation has high spectral efficiency, and, therefore, it was combined with MDM using four different modes of light operating at 80 Gbps [42] and covered 10,000 km successfully. There are two main types of light modes such as LG modes and Hermite Gaussian (HG) modes. LG modes have circular profiles, and HG modes have vertical helical rotations, and both can be generated by varying *l* and *m* numbers. HG modes were used in [43], where two 50 Gbps data streams were modulated with OFDM and carried by two different HG modes over a 20,500 km OSC link. An ultra-high capacity 4 Tbps 16,000 km OSC link at 2 µrad pointing errors was demonstrated using MDM in [44]. As researchers learned about the cutting-edge competence of light modes, they further investigated OAM beams in TLBC, such as in optical fiber communication (OFC) and optical wireless communication (OWC). The theory and literature of OAM in OWC systems are discussed in Section 1.3.

### 1.3. Introduction and Literature of OAM in OWC Systems

EM waves have four features such as amplitude, wave vector, frequency, and angular momentum (AM), and it is perceived that the AM feature of EM waves has yet to be fully explored, unlike amplitude, frequency, and wave vector. Spin AM (SAM) and OAM are the two prominent types of AM, where SAM is associated with the polarization of light and OAM with electrical field distribution around the propagation axis. OAM is preferred over SAM due to its potential to support high speed, be less prone to atmospheric effects, and offer mathematical encryption of data which in turn provides improved security. Ring-shaped and helical structures are the two types of OAM beams generated from the direction of rotation of phase and intensity patterns. Different OAM beams can be generated by changing the charge value (*l*), and the expression for OAM is given as $\Psi \left(r,\phi \right)=A\left(r\right)\frac{\left(e^{il\phi }\right)}{\sqrt{2\pi }}$ [45], where $A\left(r\right)$ is the radial basis set and ϕ is the azimuthal angle. OAM is compatible with WDM, TDM, and OCDMA, which makes it a more attractive data transmission multiplexing technique. A 20 Gbps OTC link with different OAM beams having values of *l* = +1, +3 was demonstrated using 4 × 4 multi-input multi-output (MIMO) technology [46]. A high-capacity WDM-OAM system was presented in unmanned aerial vehicle communication at 40 Gbps [47]. A high speed of 39.06 Gbps was successfully achieved in an OTC system employing OAM beams [48], and further index modulation was discussed in [49]. In 2022, an OTC system at 40 Gbps is reported using NRZ, RZ, and alternate mark inversion (AMI) modulation employing LG00, LG130, LG400, and LG800 OAM beams [50]. The results revealed that NRZ performed best in terms of BER, and the further research conducted in [51], NRZ was replaced with PDM-QPSK, and it was seen that the data rate supportability increased 10 times, i.e., 400 Gbps. In OWC systems, OAM beams are not investigated widely and are either limited to theoretical proposals [52] or restricted to lower earth orbit (LEO) based communications [53]. Moreover, mode crosstalk is prevalent in reported OTC systems because they have used consecutive LG/HG mode profiles. For example, LG00 and LG01 modes were used in a 2 × 40 Gbps MDM-IsOWC system [1], HG00 and HG01 modes were incorporated in 100 Gbit/s CO-OFDM-MDM-IsOWC system, HG00 and HG01 modes were used in 10 × 400 Gbps Is-OWC system [44], LG11 to LG15 (consecutively), and HG11 to HG15 (consecutively) in 120 Gbps optical code division multiplexed IsOWC link [54]. Thus, this research work is focused on designing ultra-high capacity and long-reach OAM-OSC systems employing mode crosstalk-suppressed OAM multiplexing by selecting distant LG modes.

### 1.4. Limitations in OAM-Based OWC Systems

Despite the numerous advantages of OAM in OWC systems, there are some predominant limitations such as (1) mode crosstalk in OAM beams due to smaller differences in l between different modes, (2) lower power efficiency of the receiver due to very high order LG modes in OAM, (3) mode leakage due to pointing errors, and (4) phase modulation under atmospheric turbulences. As per the author’s best knowledge, OAM beams are not investigated in OSC systems for ultra-high capacity and long transmission.

In this work, an ultra-high capacity OSC system is presented incorporating the OAM multiplexing technique over 22,000 km, and a detailed investigation of the OSC system is performed at different input parameters such as OSC link length, pointing errors, and receiver telescope antenna diameter in terms of log BER, signal to noise ratio (SNR), and EVM%.

The rest of the paper is structured as follows: Section 2 discusses the principle of OAM and OSC. The simulation of the proposed OAM-OSC system is elaborated in Section 3. The investigation and results of the proposed system are given in Section 4, followed by a state-of-the-art comparison with reported OSC systems and with final concluding remarks in Section 5, along with the future scope.

## 2. Principle of OSC Systems and LG Modes

Compared to RFC, large bandwidth and high speed are offered by OSC due to the incorporation of high-frequency optical carriers, and these features make OSC a paramount data transmission candidate for space-based satellite communications. Moreover, high frequency in OSC enables manufacturers to design lightweight satellites due to smaller size antennas.

### 2.1. Principle of OSC Systems

For the long-distance data transmission between two satellites, high-power signals are transmitted ($T_{P}$) towards the receiver, and receiver power (*R_P_*) is expressed as:

(1) $$R_{P}=T_{P}T_{E}R_{E}(\frac{\lambda }{4\pi L}{)}^{2}T_{G}R_{G}T_{PL}R_{PL}$$

It is depicted in Equation (1) that $R_{P}$ depends on different satellite parameters such as the optical efficiency of the transmitter ($T_{E}$) and receiver $R_{E}$, wavelength of operation λ, transmitter and receiver telescope gain denoted by $T_{G}$ and $R_{G}$ respectively, and loss due to pointing errors in the transmitter $T_{PL}$, and in the receiver $R_{PL}$. $T_{G}$ and $R_{G}$ are calculated as $T_{G}={\left(\frac{\pi D_{TTS}}{\lambda }\right)}^{2}$, and $R_{G}={\left(\frac{\pi D_{RTS}}{\lambda }\right)}^{2}$, respectively, where TTS is used for the transmitter telescope, RTS for the receiver telescope and D is the telescope diameter. Narrow beam-based lasers are employed in OSC communication because they are coherent, directional, and have the potential to cover prolonged distances. However, PTE in OSC are two of the maximum performance deteriorating issues and in particular, a smaller field of view (FOV) of the receiver introduces maximum signal loss due to misalignment between transmitter and receiver satellite antennas. Therefore, $T_{\theta }$ is azimuth pointing error angle in the transmitter and $R_{\theta }$ in receiver, and $T_{PL}$ and $R_{PL}$ are expressed in Equations (2) and (3), respectively, as [55]:

(2) $$T_{PL}=e^{-T_{G}T_{\theta }^{2}}$$

(3) $$R_{PL}=e^{-R_{G}R_{\theta }^{2}}$$

For the realization of an ideal OSC system, as observed from Equations (1)–(3), $T_{PL}$ and $R_{PL}$ should be minimum, and $T_{G}$ and $R_{G}$ along with $T_{E}$ and $R_{E}$ should be maximum.

### 2.2. LG Modes for OAM Generation

**Figure 2:**
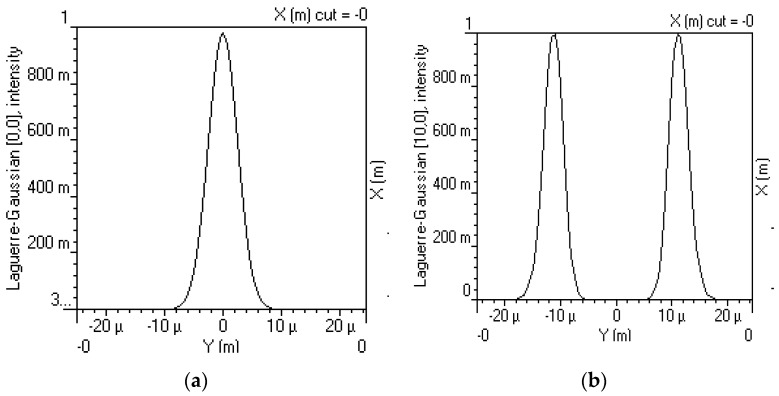
Different LG mode profiles at (**a**) l=0, m=0 and (**b**) l=10, m=0 in 2D.

There are two basic phenomena in light propagation through free space, such as (1) with the propagation of light in OWC, a significant change in the intensity profiles of light has been observed; and (2) modes of light (i.e., electrical field distribution) cannot change with the transmission through free space. The direction of the variation in the electrical field remains constant only; however, other components such as phase, profile, and receiver power of light keep on changing. The generation of LG and HG modes is the outcome of constant electrical field distribution of light in wireless mediums. Both LG and HG modes differ in structure shape such as LG modes have a horizontal circular structure and HG modes have a vertical helical structure. Preferred modes for the communication systems and OAM are LG modes due to their simple multiplexing/de-multiplexing and can have a significant gap in the rings depending upon *l*. The LG mode profile depends on the focus point, the axis of the beam, the optical frequency, and the radius of the beam. Doughnut, like the shape of LG modes, has a helical phase front and it is expressed as [56]

(4) $$\Psi_{lm}\left(r,\emptyset \right)=(\frac{2r^{2}}{w_{0}{}^{2}}{)}^{\left|l\right|}L_{m}^{l}\left(\frac{2r^{2}}{w_{0}{}^{2}}\right)\exp\left(\frac{r^{2}}{w_{0}{}^{2}}\right)\exp\left(j\frac{\pi r^{2}}{\lambda R_{0}{}^{2}}\right)\{\begin{array}{c} \cos\left(\left|l\right|\phi \right),l\geq 0\\ \sin\left(\left|l\right|\phi \right),l<0 \end{array}$$

where X and Y indices are the l,m indexes, light profile radius R, spot size $w_{0}$, Laguerre polynomial $L_{lm}$, z direction spot size $w\left(z\right)$. Diverse profiles of light can be generated in LG modes by changing the l,m numbers, for example, LG00 is generated by fixing l,m at l=0,m=0, and LG100 is obtained by entering l=10,m=0 as shown in Figure 2.

The number of peaks in the light profile is a function of *l* and the boundary around these peaks varies with *m.* These different LG modes with distant values of *l* are combined to realize OAM multiplexing for high-speed OWC systems.

## 3. Simulation Design of Proposed PDM-256-QAM Modulated OAM-OSC System

**Figure 3:**
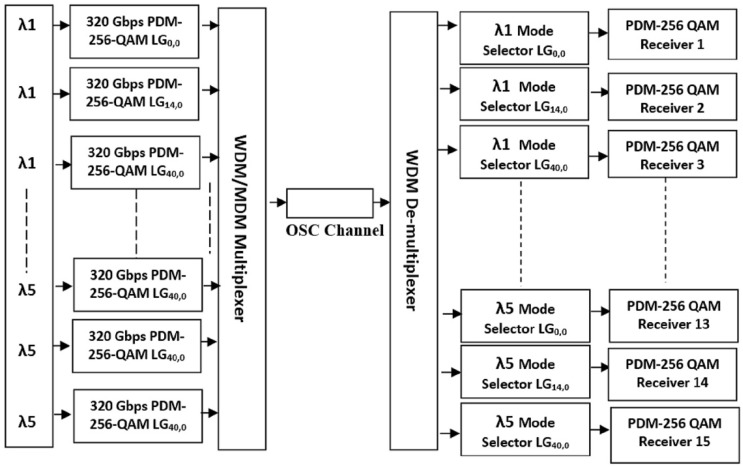
System setup of proposed 320 Gbps × 5λ × 3 OAM modes based OSC system.

For OSC systems, single polarization QAM [57] and dual polarization QAM with different variants such as 4-QAM [37], 16-QAM [58], 64-QAM [59], and 128-QAM [60] have been reported several times and revealed that bandwidth efficiency of higher QAMs is better than conventional QPSK, DPSK, etc. modulations. Therefore, we employed dual polarization 256-QAM in our system. Figure 3 represents the block diagram of the proposed 4.8 Tbps OSC system using PDM-256-QAM and hybrid WDM-OAM multiplexing. A data rate of 320 Gbps is transmitted over each channel, and there are a total of 5 wavelengths, but a total of 15 channels are generated such that each wavelength is used thrice in the system. Three different LG modes are considered for each wavelength and distributed in a manner that three channels of the same wavelength receive different LG modes, e.g., LG0,0 for the first channel, LG14,0 for the second channel, and LG40,0 for the third channel in the case of all 5 wavelengths. In this way, the cost of multiple lasers is reduced by 1/3rd using different LG modes that enable us to realize 15 channels from 5 wavelengths only. All the channels are modulated with PDM-256-QAM multilevel modulation due to its narrow spectrum, high tolerance against dispersion, and minimum level noise.

**Figure 4:**
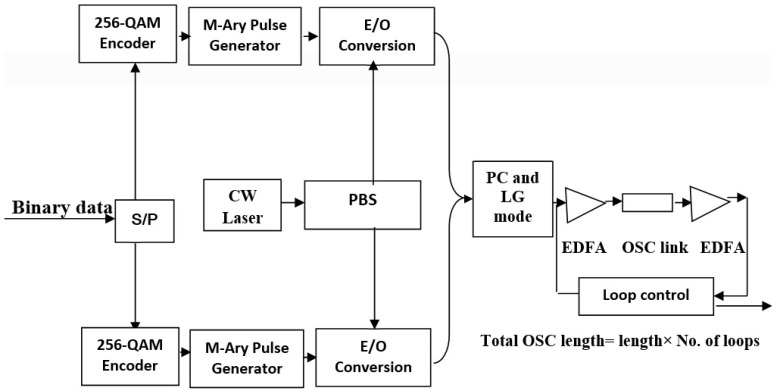
Internal structures of PDM-256-QAM transmitter and OSC loop control.

As shown in Figure 4, first and foremost, a binary data stream at the rate of 320 Gbps is generated from the BER test set, followed by a serial to parallel converter (S/P). The major function of S/P data conversion is to provide bandwidth efficiency such that the bandwidth required for the transmission of serial data is higher than the parallel data bits.

A symbol rate of 20 Gsymbols per second is achieved using 8 bits per symbol in the QAM, making it 256-QAM. The M-ary pulse generator provides m number of different levels to different 8 bits per symbol and for the generation of different polarizations in the system, laser signals are divided into two different SOPs incorporating polarization beam splitter (PBS) such that horizontal and vertical polarizations can be realized. Further, these horizontal and vertical SOP-based laser signals are given to the upper and lower Mach–Zehndar modulators, respectively. Further, data modulated on these two SOPs are combined with the help of a polarization controller (PC) and a particular LG mode is assigned to each channel. To accommodate all the channels, a WDM multiplexer is employed for different wavelengths, and an MDM multiplexer is incorporated to realize OAM multiplexing from different LG modes. WDM-OAM multiplexed signals are communicated over an OSC loop having an OSC channel, and pre and post-erbium-doped fiber amplifier (EDFA). Simulation specifications are listed in Table 1.

**Figure 5:**
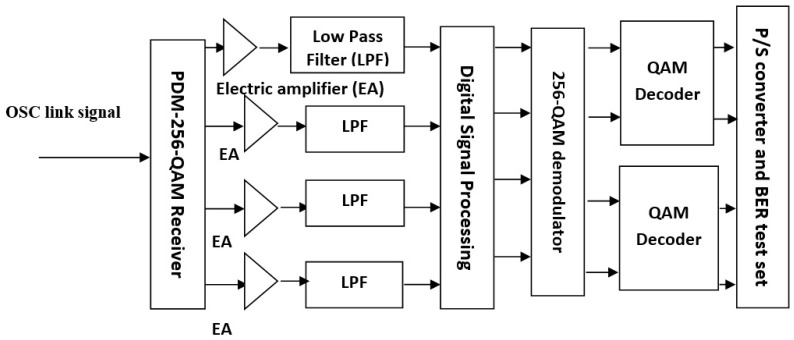
Components of the PDM-256-QAM receiver.

**Figure 6:**
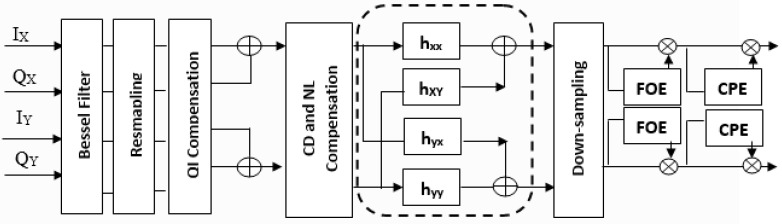
DSP module with 8 functions, pre-processing, and different algorithms.

Signals after traveling through the OSC channel reached the receiver section placed in the destination satellite. A de-multiplexer 1 × 5 is placed to route all five wavelengths to the specific port and further, each wavelength is divided into three sub-channels. OAM beams are filtered at each sub-wavelength and followed by a PDM-256-QAM receiver as shown in Figure 5. Balanced detection employing PIN photodetectors, and phase synchronizations using local oscillators (LOs) are performed followed by electrical signal amplification. DSP module in the next section compensates for the performance deteriorating effects such as nonlinear effects, chromatic dispersion (CD), in-phase quadrature-phase (IQ) compensation, phase errors, etc., as illustrated in Figure 6. BPS, VPE, and CMA are the three important algorithms operating in DSP. A total of 256 symbols at different amplitudes and phases are then demodulated with a 256-QAM decoder, followed by the extraction of binary bits stored in the symbols. Binary data is in parallel form and then converted into a serial data stream. The BER test set in the end component compares the received and transmitted binary data bits and provides results in terms of BER at each polarization.

## 4. Investigation of Proposed OAM-OSC System at Different Input Parameters

**Figure 7:**
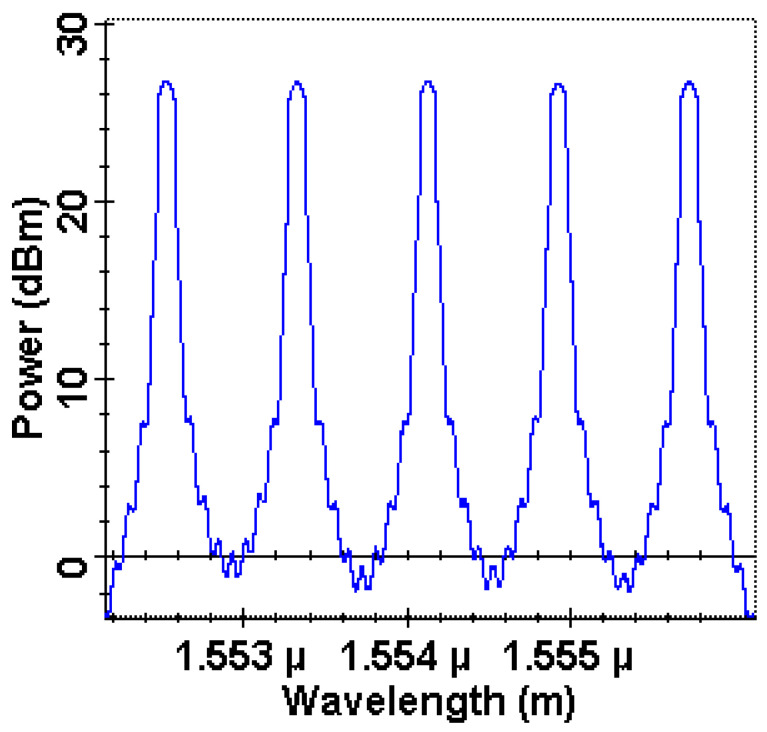
Representation of OCSs of different wavelengths.

This section covers the detailed performance analysis of the proposed system at different input parameters such as distance between satellites, pointing errors, and receiver antenna diameters, and we further explore the performance of each OAM beam in the system in terms of BER, EVM, and constellations. For the investigation of the proposed OAM-OSC system, a prominently used simulation environment, Optisystem, is considered. Figure 7 illustrates the presence of five different wavelengths in the system after the WDM multiplexer in the c-band has a power of 26.7 ± 0.05 dB. Checking of OCSs after regular intervals is required to see the signal availability and any unusual drop or significant power loss that indicates the link breakdown or any obstruction between the transmitter and receiver.

**Figure 8:**
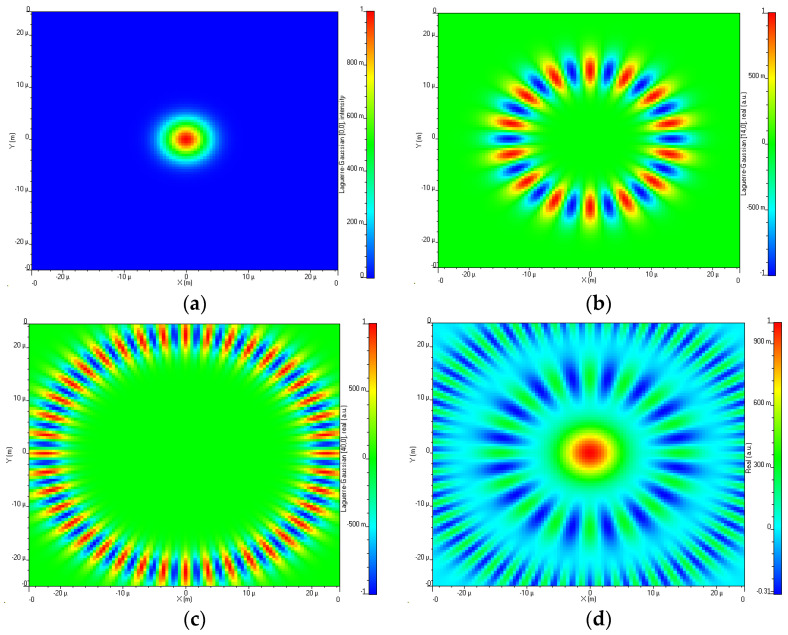
Transverse mode profiles (**a**) LG0,0, (**b**) LG14,0, (**c**) LG40,0, and (**d**) OAM multiplexed beams.

Light profiles of different LG modes such as LG0,0, LG14,0, and LG40,0 are represented in Figure 8a–c followed by the OAM multiplexed modes in Figure 8d.

We have considered an appropriate (optimal) difference in l so that only minimum mode coupling occurs and is restricted to a maximum value of 40 because detection becomes difficult at higher-order modes.

**Figure 9:**
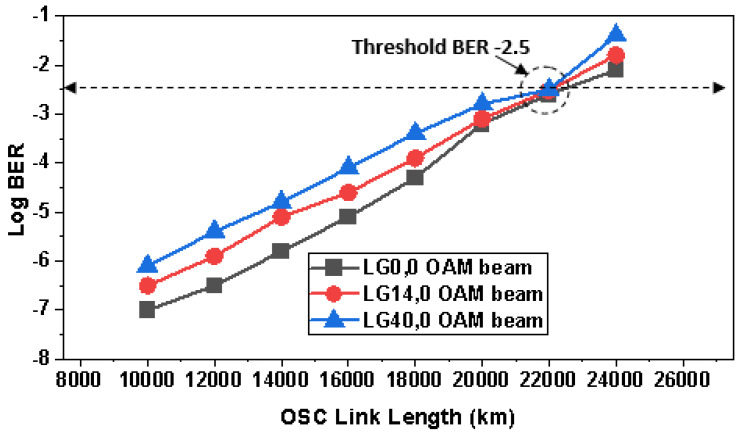
Variation of BER for OSC link length for LG0,0, LG14,0, and LG40,0 OAM beams.

The majority of satellite communications take place in space, where there is no atmosphere and only a vacuum exists, resulting in less power loss for the signal. However, the distance between satellites is the major factor that derives the final performance of received signals. Therefore, the OSC link length is increased from 10,000 to 24,000 km in the proposed investigation to find out the maximum supported distance by OAM-OSC. BER is validated for different OAM beams at different OSC links as shown in Figure 9.

With the increase in the OSC link length, symbols at the receiver deteriorate and displace from their ideal positions, which in turn increases the BER. Furthermore, phase errors in the 256-QAM symbols, attenuation, scattering, and nonlinear effects at high power also introduce bit errors. It is discerned that all the OAM beams have different output BER such that fundamental mode LG0,0 has the least BER due to narrow beam mode and LG40,0 has the highest BER because of the power losses at the receiver due to higher order mode. In this case, the receiver antenna diameter is 15 cm, and therefore, small-size antennas are sometimes not wide enough to accumulate all the power of higher-order modes. However, all the OAM beams have covered 22,000 km successfully within the acceptable log BER condition i.e., −2.42 [40].

**Figure 10:**
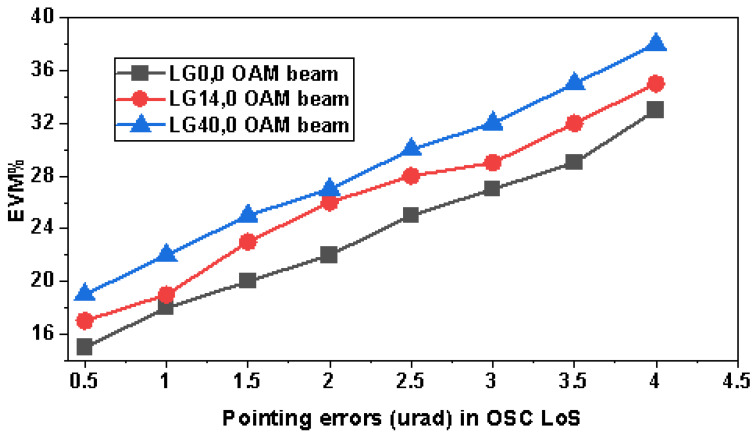
Performance of OAM beams in OSC link having different pointing errors at 10,000 km.

Relay communication in OSC systems plays an important part in covering ultra-long transmission distances between satellites, and LoS is the utmost condition to be satisfied. Slight misalignment in the LoS can cause severe bit errors and performance degradation. Moreover, in the case of OAM beams, the spot size of LG modes increases with the distance, and slight misalignment can cause high mode power loss due to the small diameter of the OSC receiver telescope. Therefore, in Figure 10, we investigated the effects of different pointing errors on OAM beams in terms of EVM%. Maximum and minimum pointing errors investigated are 0.5 to 4 µrad and it is observed that the lowest spot size of LG0,0 experienced the lowest symbol deterioration due to the highest RAP and, in turn, provided the lowest EVM%. The value of different EVM% for LG0,0 is 15.01, for LG14,0 is 16.99, and for LG40,0 is 19 at 0.5 µrad, and for increased pointing errors such as 4 µrad, EVM% increased to 32.98, 35.02, and 37.99 for LG0,0, LG14,0, and LG40,0 respectively. Higher-order modes have very wide spot sizes of light profiles, and it is tedious for the receiver antenna to detect the entire mode power.

**Figure 11:**
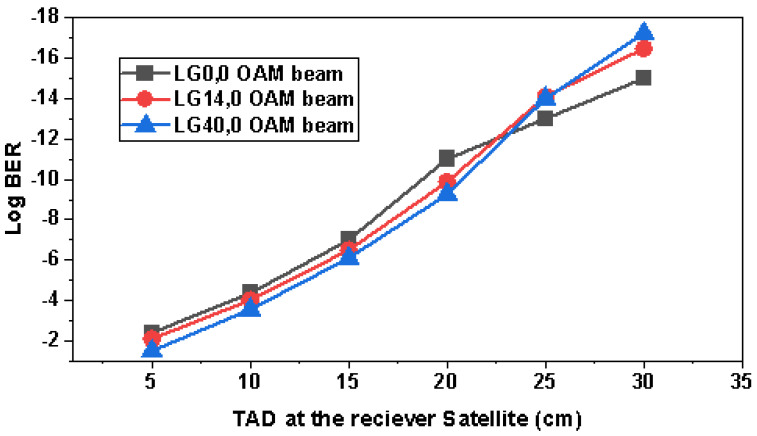
Log BER variation with different TADs at the receiver satellite.

Figure 11 illustrates the effects of the receiver satellite incorporated telescope aperture diameter (TAD) on the log BER of different OAM beams. The results revealed that the higher the diameter of the receiver satellite antenna, the more power is accumulated at the receiver and the better the log BER. It is interesting to observe that until the TAD of 20 cm, LG40,0, and LG14,0 OAM beams experienced more log BER as compared to LG0,0, but at TAD values greater than 20 cm, LG40,0 and LG14,0 OAM beams surpassed the performance of LG0,0 due to more RAP at the receiver. Moreover, LG40,0 provides the least log BER (−17.23) followed by LG14,0 (−16.47) at 30 cm TAD at the receiver satellite, and maximum log BER is observed in the case of LG0,0 (−15). whereas LG0,0 was encountering the least BER (−2.4) at 5 cm TAD and LG40,0 had the highest (−1.5) bit errors. It is suggested to use higher TADs for the higher-order OAM beams.

**Figure 12:**
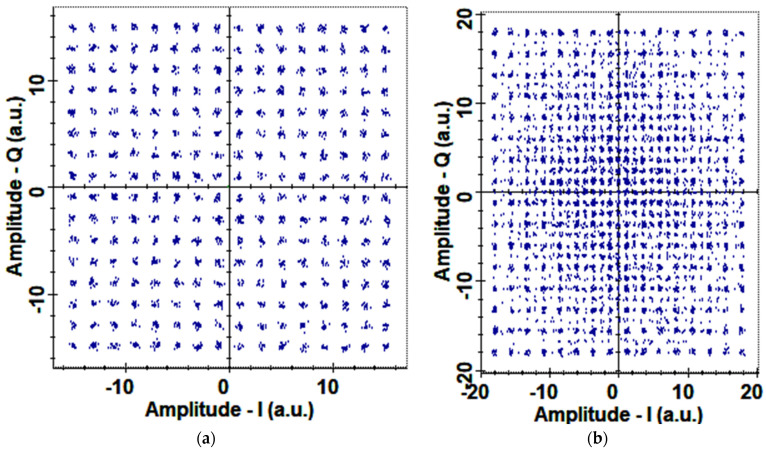
A 2D plot of 256-QAM symbols on the I and Q axis at OSC link length of (**a**) 22,000 and (**b**) 24,000 km.

The proposed OSC systems have PDM-256-QAM modulation that has 8 bits per symbol and in turn, generates 256 different symbols in the data transmission. These 256 symbols have a fixed phase and amplitude at the transmitter, and due to noise and other performance-limiting effects, their phase and amplitude become distorted as they travel toward the receiver. At the receiver, optical signals are converted into electrical data with balanced detection, and these 256 symbols are represented by a constellation analyzer. A constellation analyzer is a two-dimensional (2D) plot with an I and Q axis for the symbols received at the receiver. EVM can also be calculated from the constellation analyzer as the displacement of symbols from their ideal positions can be seen from the 2D plot. Figure 12a depicts the constellation diagram for PDM-256-QAM at 22,000 km OSC link distance using DSP and it is evident that all the symbols have little effects of noises but, despite that are at their respective positions. On the other hand, at the OSC link length of 24,000 km, there are high distortions in the symbols on the I and Q axis as shown in Figure 12b.

**Figure 13:**
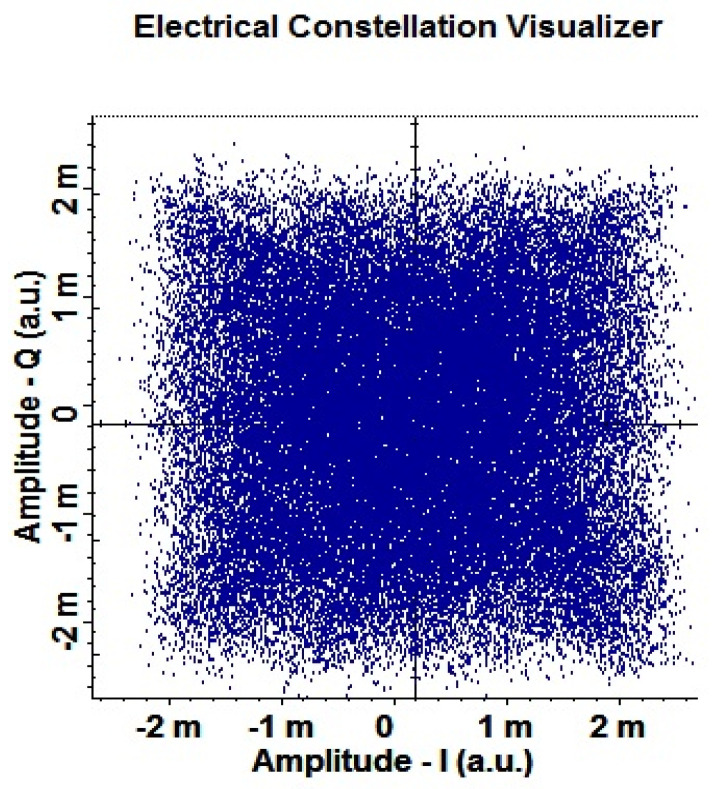
Highly distorted constellation of 256-QAM symbols at 22,000 km without DSP.

Further, we explored the effects of DSP on the OAM-based OSC system and it was observed that DSP had a significant impact on the performance of the systems due to the compensation of NL, dispersion, phase errors, etc. Figure 13 illustrates the constellation diagram of the proposed OAM-OSC system at 22,000 km without using DSP. Results are completely distorted without DSP, and if DSP is incorporated, then the constellation gets improved as depicted in Figure 12a at the same OSC distance.

**Figure 14:**
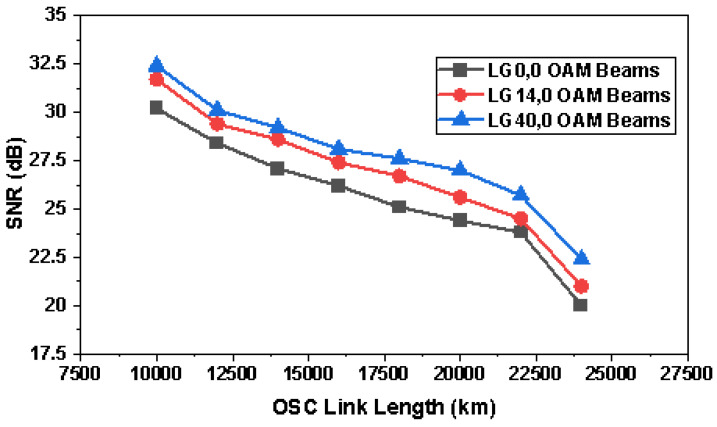
Representation of SNR versus OSC link lengths in the case of three OAM beams.

Figure 14 represents the performance of the proposed system at OSC link lengths for different OAM beams and it is observed that SNR reduces as the link length prolongs. The performance of LG00 OAM beams was found to be best performing and LG400 exhibited the least SNR.

### 4.1. A Detailed Comparison of Proposed OAM-OSC System with Reported OSC Systems

In this section, the performance of the proposed OAM-OSC system is compared with the different reported OSC systems in terms of data rate, channels, capacity, OSC link length, modulation used, multiplexing employed, and performance, as shown in Table 2. It is observed that the highest capacity is accomplished in the proposed work as compared to [41,42,44,61,62,63]. Moreover, the use of DQPSK [41], 4-QAM/PSK-OFDM [43], PDM-QPSK [44], SP-QAM [59], PDM-4-QAM [37], PDM-16-QAM [58], PDM-64-QAM [59], and PDM-128-QAM [62] have been reported in these researches. However, the proposed system has employed high spectral efficiency (eight bits per symbol) based PDM-256-QAM in OSC-OAM system. As far as multiplexing is concerned, MDM was used by [41,43,44], but due to the consecutive or lesser differences in LG/HG mode profiles, more deterioration and limited reach were obtained. On the other side, the proposed OAM-enabled OSC system with PDM-256-QAM offered the highest capacity, ultra-long reach, and high cost-effectiveness using single wavelength thrice for data modulation with OAM beams.

### 4.2. Contributions of Proposed OSC-OAM System

We investigated a PDM-256-QAM-based OSC system incorporating three different OAM profiles for the first time. A detailed literature review made us understand the shortcomings of OSC systems, such as low capacity, shorter OSC link length, high cost, mode crosstalk, and high BER. The performance of the OSC systems depends on different parameters such as pointing errors, LoS, transmitter power, antenna aperture size, antenna gain, beam divergence, etc. However, the two major performance-affecting entities are modulation and multiplexing. For a high bandwidth efficient carrier spectrum, PDM-256-QAM was considered in the proposed work, and for reducing mode crosstalk, OAM is selected. As explained earlier, the most significant limitation encountered in most of the MDM-OSC systems is the multiple modes placed with shorter gaps [1,43,44,56], and this issue led us to use OAM beams with a larger gap between LG modes. The cost of the system is a key concern where multilevel modulations such as QPSK, QAM, and OFDM are employed. Therefore, we employed a single laser for different three channels through three different OAM beams and reduced the cost of the system by one third. Further investigation of the proposed OSC-OAM system revealed some significant findings, such as the fact that higher LG modes require wider receiver TADs for better performance and that basic mode profiles, such as LG00, provide the best performance.

## 5. Conclusions

In this research study, an ultra-high capacity OSC system was presented over a 22,000 km link distance between satellites, and we have also manifested the potential of OAM beams by incorporating LG00, LG140, and LG400 transverse mode profiles and PDM-256-QAM. OAM beams were selected at an optimal one number difference to reduce the effects of mode coupling and to avoid generating very higher order modes, which in turn reduce RAP at the receiver. A total of 15 channels were generated from only 5 wavelengths using LG modes, which reduced the cost of the system by one third. Results revealed that all the OAM beams had different output BER at diverse OSC link lengths such that the fundamental mode LG0,0 had the least BER due to the narrow beam mode spot size and LG40,0 had the highest BER because of the power losses at the receiver due to the higher order mode. The EVM% value for LG0,0 was 15.01, for LG14,0 was 16.99, and for LG40,0 was 19 at 0.5 µrad, and for increased pointing errors such as 4 µrad, EVM% increased to 32.98, 35.02, and 37.99 for LG0,0, LG14,0, and LG40,0, respectively. Higher-order modes have very wide spot sizes of light profiles, and it becomes tedious for the receiver antenna to detect the entire mode power. Therefore, it is interesting to observe the effects of different TADs. It is perceived that until the TAD of 20 cm, LG40,0 and LG14,0 OAM beams experienced more log BER as compared to LG00, but at TAD values greater than 20 cm, LG40,0 and LG14,0 OAM beams surpassed the performance of LG0,0 due to higher RAP at the receiver. Moreover, LG40,0 provided the least log BER (−17.23) followed by LG14,0 (−16.47) at 30 cm TAD at the receiver satellite, and the maximum log BER was observed in the case of LG0,0 (−15). Whereas LG0,0 encountered the least BER (−2.4) at 5 cm TAD and LG40,0 had the highest (−1.5) bit errors. It is suggested to use higher TADs for the higher-order OAM beams. We considered LG mode profiles with larger differences such as LG00, LG140, and LG400 to realize OAM multiplexing, and it was observed that mode coupling becomes insignificant in these distant mode profiles as compared to the reported research where LG/HG modes with lesser gaps are considered [1,43,44,58]. We performed an extensive literature survey and found that the highest capacity that has been reported so far in OSC is 4 [44], 3.84 [61], 1.6 Tbps [62], and 320 Gbps [63]. Therefore, an ultra-high capacity 4.8 Tbps OSC-OAM system was presented for the first time in this work. Further, we explored the effects of DSP on the OAM-based OSC system and it was observed that DSP had significant effects on the performance of the systems due to the compensation of NL, dispersion, phase errors, etc.. In the end, a state-of-the-art comparison revealed that the proposed OAM-OSC system is better than reported OSC systems, as per the authors’ best knowledge. In the near future, more OAM beams can be incorporated into the system.

## Figures and Tables

**Table 1:** Simulation parameters of proposed OAM-OSC system.

Parameter	Values
System capacity	4.8 Tbps
Wavelengths and channel spacing	5 (1552.52, 1553.32, 1554.12, 1554.92, 1555.72) and 0.8 nm
Total channels	15
Laser Input Power and Extinction ratio (ER)	30 and 30 dB
Modulation	PDM-256-QAM
OSC length	10,000 km to 24,000 km
OSC wavelength window, transmitter, and receiver antenna diameter	C band, 1–5 15 cm
Transmitter and receiver optical efficiency and Pointing errors	0.8 and 1 µrad
Symbol rate and sample per bit	20 Gsymbol/s and 8
OAM beams	LG0,0, LG14,0, and LG40,0
EDFA gain and noise figure in pre/post	20, 4 dB
Photodetector and responsivity	PIN and 0.8 A/W
DSP algorithms for dispersion, phase, and nonlinear effects compensation	BPS, CMA, VPE

**Table 2:** Detailed comparison of proposed OAM-OSC and reported OS systems.

Parameters	H.K. Gill et al. (2019) [41]	K. Singh et al. (2021) [43]	S. Chaudhary et al. (2022) [44]	Proposed OAM-OSC System
Data rate	10, 20, 40 Gbps	50 Gbps	400 Gbps	320 Gbps
Channels	64	2	10	15
Capacity (max.)	2.5 Tbps	100 Gbps	4 Tbps	4.8 Tbps
OSC Link Length	3750 km	20,500 km	16,000 km	22,000 km
Modulation	DQPSK	4-QAM/PSK-OFDM	PDM-QPSK	PDM-256-QAM
Multiplexing	MDM	MDM	MDM	OAM
BER	10^−9^	10^−3^	10^−3^	10^−3^

## Data Availability

No new data were created or analyzed in this study. Data sharing is not applicable to this article.
